# Peculiar Form of Dissecting Aneurism of the Thoracic Aorta

**Published:** 1845-06

**Authors:** 


*Peculiar form of Dissecting Aneurism of the Thoracic Aorta.*—Dr. Robert Macdonnell, in the last number of the Medical Gazette, relates a case of this unusual form of aneurism. The patient, a women, about fifty years of age, went to bed in perfect health, and towards morning was awoke by very severe pain in the epigastric region, resembling colic, to attacks of which she was subject. An anodyne draught was administered, and warm fomentations to the abdomen. In an hour afterwards she suddenly expired.

On a post-mortem examination the pericardium was found to contain about four ounces of serum tinged with blood, and some coagula; the membrane was otherwise healthy. The origin of the great vessels, towards the apex of the pericardium, was surrounded by a large and firm mass of coagulated blood, which was bound down by the thin layer of serous membrane which passes up from the heart along the vessels to be reflected on the fibrous layer. The semilunar valves of the aorta were atrophied and cribriform; the aorta at this place was healthy, but about an inch from it, a laceration, extending transversely, was discovered, the edges well defined, which penetrated the internal and middle coats only; from it a probe could be passed downwards between the external and middle coats to the level with the upper border of the semilunar valves. A probe passed upwards, as far as the division of the innominata, and for half an inch along the course of the left subclavian and carotid, to which extent the middle coat of these vessels was separated from the external.

The opening in the cellular coat through which the blood escaped was small and circular; it was filled with a dark coagulum which extended downwards; separated the aorta from the pulmonary artery, and its compression had occasioned flattening of this vessel. "The coagulum lay beneath all that portion of the reflected layer of the pericardium, extending from the zonæ tendinæ of the right and left ventricles to where it is reflected on the under surface of the fibrous layer of the membrane. The serous membrane was perfectly whole, except at a small point corresponding to the junction of the right ventricle with the left auricle, where there was a small aperture, through which the small quantity of blood in the bag of the pericardium had evidently escaped."

The aorta itself was extensively diseased, and thickly coated with bony plates and atheromatous deposit. The mouth of the innominata was filled by a firm clot, which extended for some distance along the vessel. The lungs and liver were greatly engorged.—*Dublin Medical Press.*

